# Research Priorities to Improve the Management of Acute Malnutrition in Infants Aged Less Than Six Months (MAMI)

**DOI:** 10.1371/journal.pmed.1001812

**Published:** 2015-04-21

**Authors:** Chloe Angood, Marie McGrath, Sagar Mehta, Martha Mwangome, Mary Lung’aho, Dominique Roberfroid, Abigail Perry, Caroline Wilkinson, Anne-Dominique Israel, Cecile Bizouerne, Rukhsana Haider, Andrew Seal, James A. Berkley, Marko Kerac, MAMI Working Group Collaborators

**Affiliations:** 1 Emergency Nutrition Network, Oxford, United Kingdom; 2 Washington University School of Medicine, St Louis, Missouri, United States of America; 3 KEMRI-Wellcome Trust Research Programme, Kilifi, Kenya; 4 CARE, Atlanta, Georgia, United States of America; 5 Nutrition and Child Health Unit, Institute of Tropical Medicine, Antwerp, Belgium; 6 Department for International Development, London, United Kingdom; 7 Public Health Section, Division of Programme Support and Management, United Nations High Commissioner for Refugees, Geneva, Switzerland; 8 Action Contre La Faim, Paris, France; 9 Training & Assistance for Health & Nutrition (TAHN) Foundation, Dhaka, Bangladesh; 10 Institute for Global Health, University College London, London, United Kingdom; 11 Nuffield Department of Medicine, University of Oxford, Oxford, United Kingdom; 12 Leonard Cheshire Disability & Inclusive Development Centre, University College London, London, United Kingdom; 13 London School of Hygiene & Tropical Medicine, London, United Kingdom

## Abstract

By engaging expert opinion, Marko Kerac and colleagues set research priorities for the management of acute malnutrition in infants.

**Summary Points**

- Worldwide, 8.5 million infants aged less than 6 months (<6m) are acutely malnourished. For the first time, 2013 WHO Malnutrition Guidelines describe their treatment, but on the basis of “very low quality” evidence, per WHO. More and better research is urgently needed.
- To prioritise the many possible research questions on infant <6m malnutrition, we used the systematic, transparent, well-established Child Health and Nutrition Research Initiative (CHNRI) approach. Sixty-four experts scored 60 research questions on the basis of their answerability, likelihood of intervention efficacy, effectiveness, deliverability, sustainability, impact on disease burden, and impact on equity.
- “How should infant <6m SAM be defined?” was the top-scoring research question; that this and other basic questions are still needed highlights paucity of evidence on this topic.
- Other leading questions reflect interest in public health/community-focused models of care, e.g., “What are priority components of a package of outpatient care?” These questions are important to inform new outpatient strategies now recommended by WHO.
- Most of our questions received high-priority scores reflecting a great need for a wide variety of evidence. Several major global initiatives such as the “Scaling Up Nutrition Movement” and “Generation Nutrition” would benefit from better evidence. Our results show clear ways forward for future research investments.

## Background

Undernutrition is responsible for some 3.1 million child deaths per year, 45% of all deaths among children aged <5 years [1]. Infants are most at risk [2,3]. Acute malnutrition (comprising wasting, as defined by low weight-for-length; and/or low mid-upper arm circumference; and/or oedematous malnutrition, Kwashiorkor) is particularly important because of its high case fatality rate [4,5].

In the last decade, the management of acute malnutrition has been revolutionized by a new public health-orientated approach to treatment, “Community-based Management of Acute Malnutrition” (CMAM) [6]. To achieve impact, CMAM focuses on: high programme coverage; treatment of “uncomplicated” cases on an outpatient basis; the use of nutrient-dense “ready-to-use therapeutic food” [7]. Programmes are targeted at children aged 6 to 59 months. Contrasting this success, the management of acute malnutrition in infants under six months old (<6m) is often neglected in practice even if described in clinical protocols [8]. When it is described, guidelines focus on inpatient-based care alone [9]. Yet, recent estimates suggest that an estimated 3.8 million infants <6m have severe acute malnutrition (SAM) [10]. This is a minimum estimate since it does not include oedematous SAM. A further 4.7 million infants are estimated to have moderate acute malnutrition (MAM) [10].

Recently updated (December 2013) World Health Organization (WHO) guidelines on the “Management of Severe Acute Malnutrition in Infants and Children” for the first time have a chapter devoted to infants <6m [11]. Other key firsts in these guidelines are: distinguishing between “complicated” (i.e., clinically sick) and “uncomplicated” (i.e., malnourished but clinically stable and able to eat) cases of infant <6m SAM; outlining outpatient as well as inpatient-based treatment options [11]. This represents significant progress. However, whilst many of the WHO recommendations on infants <6m are “strong” [12], the quality of evidence underlying them is “very low” [11,12]. The need for research is widely recognised [11,13,14]. Given that many research options (questions) are possible and that undertaking any of them requires significant investment of time and resources, it is vital to concentrate on the most important questions. A research prioritisation exercise focused on infants <6m is especially timely, relevant, and useful because:

- Nutrition plays a key role in the 2015 Millennium Development Goals (MDGs) [15] and will continue to matter beyond 2015 [16,17].
- In May 2014, a multi-agency global campaign, “Generation Nutrition” was launched. Its ultimate aim is to “End child deaths from acute malnutrition” [18].
- In November 2014, the first ever “Global Nutrition Report” highlighted a lack of progress on ending child wasting. It also noted the need to improve coverage of SAM treatment services [17].
- The “Scaling Up Nutrition” (SUN) Movement is a rapidly expanding global movement with 54 countries currently signed up [16]. It focuses on the first 1,000 days of life as a critical window of opportunity to improve nutrition; malnourished infants <6m fall within the movement’s scope.

We used the well-established Child Health and Nutrition Research Initiative (CHRNI) methodology [19] to consult key stakeholders on a range of research questions in order to identify the most important. We intend this data to be useful in informing the research agenda of governments, researchers, investors, international organisations, and national agencies about what is likely to result in high-impact policies and practices in the management of acute malnutrition in infants <6m (MAMI) in resource-poor settings.

## Methods

CHNRI methodology is described in detail elsewhere: on the website (http://www.chnri.org/index.php), in a methods paper [19], and in other papers using CHNRI to explore other key areas of global health [20–24]. It represents an important improvement on traditional research-priority—setting methods, such as literature searches to find gaps in research or comparing the burden of disease and estimating economic “payback,” because it is systematic and transparent about the range of possible research questions and how their final ranks have been derived. Full protocols are described in S1 Text. In brief:

We began by defining the context and criteria for priority setting. Working as a “core group” of authors of this paper, we developed a long list of possible research questions based on previous publications [8], meetings and discussions. By careful phrasing and consolidation of overlapping ideas, we edited and refined these to a manageable final list of 60 questions divided into three categories: (1) basic epidemiological research, (2) health policy and systems research, and (3) technical questions and interventions. Based on CHNRI’s conceptual framework, we also agreed on seven judging criteria (Table 1).

**Table 1:** CHNRI judging criteria for each research question (option).

CHNRI Criterion	How participants were asked to assess the criterion
**Answerability**	Would you say that a study to answer this research question is possible (e.g., feasible, ethically possible, well defined endpoints/outcomes)?
**Efficacy**	Would the intervention(s) arising from the proposed research be efficacious (i.e., under research/optimally resourced conditions)?
**Effectiveness**	Would the intervention(s) arising from the proposed research likely be effective (i.e., under routine programme conditions)?
**Deliverability**	Would the intervention arising from the research be deliverable (taking into account, for example, design, standardization, safety, health system infrastructure, human resources, and role of attitudes and demand)?
**Sustainability**	Would the interventions arising from the research be sustainable (taking into account, for example, financial affordability, adequacy of regulation, monitoring and enforcement, partnership and coordination between civil society and external donor agencies, and favourable political climate)?
**Disease Burden Reduction**	Would you say that interventions arising from the research would eventually (assuming high deliverability, affordability and sustainability) contribute to a significant reduction in infant malnutrition or mortality or morbidity?
**Equity**	Would you say that interventions arising from the research have potential to improve equity in disease burden distribution (i.e., could help all segments of the society, not just the privileged ones)?

As with other CHNRIs, it was up to individual respondents to interpret and imagine what future interventions might be and how they might meet or not meet the various criteria. We did not apply weights to these judging criteria (as some CHNRI projects have done) because infant <6m malnutrition is a new and focused area of research and we felt that un-weighted estimates would be clearer and more interpretable by our intended policy audience. Should readers using our data wish for any reason (e.g., to reflect context-specific priorities) to weight certain areas more than others, this is possible using the results spreadsheet in S1 Table and referring to CHNRI methods [19].

We distributed the list of questions and scoring criteria as an online survey to a total of 150 technical experts, experienced practitioners, and policy makers in the field of nutrition and child health (MAMI “reference group”). These were identified from participation at meetings and symposia related to nutrition and/or infant and young child feeding and represented a breadth and depth of expertise, as well as a wide spectrum of stakeholder organisations. An open invitation to participate was also posted on internet-based technical discussion forums.

Respondents applied each of the seven judging criteria to each of the 60 questions listed by a “Yes” (1 point), “No” (0 points), “Undecided” (0.5 points), or “Insufficiently informed” (missing input). Based on these responses, a research priority score (RPS) for each criterion and an overall RPS were calculated. These could range between 0% (lowest possible RPS) and 100% (highest possible RPS). The final list of priorities with criterion-specific and overall priority scores for all 60 research options is presented in S1 Table.

As well as the overall RPS given to various research options, we also assessed the level of agreement between scorers through the “average expert agreement” (AEA). This could range between 1 (perfect agreement between all respondents) and 0 (no agreement) [19].

### Ethics

As is standard for CHNRI projects, formal ethics review was not needed since the work does not involve any personal or otherwise sensitive data and deals with professional participants rather than patients, all solicited via established professional networks. Participants were aware that their responses would be used for research and publication. Those who completed the questionnaire were asked whether they were happy to be named as part of the group author list, and only those answering “yes” are listed. Individual answers to the questions are not presented and so were anonymous.

## Respondents

Sixty-four individuals participated in the survey (Table 2). They comprise the MAMI Working Group collaborating authors. Nine out of the 14 original MAMI group took part. Seven others took part anonymously. This represents a 42.6% response rate for directly invited participants. Sixty-four percent of respondents were invited directly and the rest came through posts on technical forums or word of mouth through invitees. Thirty-four respondents completed part 1 only, 20 completed part 2 only, and ten completed both parts.

**Table 2:** Profile of respondents (*n* = 64).

Main employer (*n*)	Main type of work (*n*)	Rural or Urban (*n*)	Mainly based in (*n*)
Non-governmental organisation (NGO) (27)	Operational / programme (35)	Both (42)	Europe (31)
Academic Institution (16)	Academic (19)	Rural only (16)	North America (13)
United Nations (UN) agency (8)	Policy (4)	Urban only (6)	Eastern Africa (9)
Government Institution (6)	Other (6)		South Asia (4)
Independent (7)			Southern Africa (3)
			West Africa (2)
			Central America / Caribbean (1)
			Australasia (1)

## Research Priorities

S1 Table provides the full list of questions and their scores in excel format. Table 3 shows the 15 research questions that achieved the highest overall research priority score (RPS). These achieved high scores across all of the judging criteria.

**Table 3:** The 15 research options that achieved the highest overall research priority score (%) with average expert agreement (range 0–1).

Research option (question)	Question number	Rank	Answerability	Efficacy	Effectiveness	Deliverability	Sustainability	Disease burden reduction	Equity	RPS	AEA
How should infant <6m SAM be defined?	1	1	94.3	89.5	94.1	98.3	89.7	90.4	91.7	**92.6**	**0.89**
What are/is the key opportunities/timing when infant SAM management can be incorporated with other healthcare programmes?	26	2	98.8	90.5	85.9	93.1	90.4	92.0	84.8	**90.8**	**0.79**
What are the priority components of a package of care for outpatient treatment of infant <6m SAM?	50	3	85.9	92.6	95.0	94.8	89.3	94.0	83.3	**90.7**	**0.68**
Having detected SAM in the community, what is the efficacy of providing targeted skilled breastfeeding support to caregivers of stable infants?	48	4	86.9	95.6	88.7	87.9	87.5	98.0	88.0	**90.4**	**0.57**
How can existing tools be adapted and/or linked together to better identify and manage infant <6m SAM?	29	5	91.7	88.9	87.9	91.1	96.2	94.2	81.3	**90.2**	**0.80**
What are the most feasible tools and techniques for assessing treatment programme coverage for infant <6m SAM?	14	6	89.5	86.1	88.2	100.0	92.6	84.6	82.0	**89.0**	**0.59**
What is the feasibility, effectiveness, cost-effectiveness and impact of different approaches to promote early initiation and exclusivity of breastfeeding?	58	7	91.3	95.6	86.4	91.4	88.9	86.0	83.3	**89.0**	**0.78**
What are the main barriers to existing inpatient interventions for infant <6m SAM and how might they be best addressed?	18	8	93.9	93.1	92.2	87.9	83.9	80.0	89.6	**88.7**	**0.72**
What is the effectiveness, cost, and safety of an outpatient-focused treatment model for infants with SAM?	21	9	89.2	84.3	93.3	84.5	78.8	93.8	89.6	**87.6**	**0.69**
Which supervision tools and approaches are most effective towards improving the front-line case management of infant <6m SAM?	27	10	95.0	86.8	89.7	91.4	89.3	84.0	77.1	**87.6**	**0.70**
How can existing child health and nutrition reporting systems be adapted to capture, monitor and audit data on infant <6m SAM?	28	11	92.7	91.4	80.0	96.6	92.3	83.3	76.1	**87.5**	**0.73**
What role do CMAM programmes have in delivering outpatient-based treatment for infant <6m SAM?	22	12	90.8	86.4	89.7	78.6	82.7	93.8	89.6	**87.3**	**0.71**
How does breastfeeding status and/or change in breastfeeding status impact on infant <6m SAM?	5	13	93.2	90.5	90.9	91.4	82.1	88.5	74.0	**87.2**	**0.79**
What is the coverage of existing treatment programmes for infant <6m SAM?	13	14	89.3	78.6	84.4	84.5	90.4	81.3	93.8	**86.0**	**0.84**
How can existing surveys of differing designs and at different levels be adapted to include infants <6m?	7	15	90.0	90.9	81.7	83.9	86.5	73.9	90.5	**85.3**	**0.71**

It is revealing of the current state of research around infant <6m SAM that many of the top questions are very basic. Most striking was the number-one question: “How should infant <6m SAM be defined?” Until we know how to define a problem, efforts to address it are likely to be seriously impaired. This lack of consensus on a case definition is a fundamental block in moving forward. It reflects distinguishing characteristics between infants <6m and older children regarding assessment: anthropometric measurement is more challenging [25]; some anthropometric indicators are not available (e.g., WHO weight-for-length standards do not exist for length <45 cm); clinical and feeding history matter more in infants than in older children yet can be difficult to assess. Though there is evidence that it could be successfully used [26, 27], mid-upper arm circumference (MUAC) measurement is not currently considered suitable for infants <6m. MUAC has been key for the treatment of acutely malnourished children over 6 months old since it enables detection in the community and high treatment programme coverage [28].

Other leading questions reflect an interest in public health/community approaches to infant <6m SAM management, for example, the questions ranked third, “What are the priority components of a package of care for outpatient treatment of infant <6m SAM?”; fourth, “Having detected SAM in the community, what is the efficacy of providing targeted skilled breastfeeding support to caregivers of stable infants?”; ninth, “What is the effectiveness, cost, and safety of an outpatient-focused treatment model for infants with SAM?”; and twelfth, “What role do CMAM programmes have in delivering outpatient-based treatment for infant <6m SAM?”. These rankings align well with the new WHO policy to distinguish between “complicated” and “uncomplicated” infant <6m SAM. Once classified in this way, outpatient treatment options are made possible for uncomplicated cases: details of these are vague in the WHO guidelines [11], so it is helpful that our CHNRI has given options. Outpatient care is a significant shift away from previous guidelines, which implied that all infants <6m automatically need admission [9].

The Average Expert Agreement (AEA) for the top 15 questions was overall high, indicating good agreement between the different expert respondents. The highest scoring research option also received the highest AEA score (0.89), indicating the strongest agreement about the importance of this particular research question. High overall AEA also reflects the paucity of current evidence around infant <6m SAM. Other CHNRIs have a wider distribution of RPS. With the exception of the bottom six research questions, all those we put out had an overall RPS of 70% and above. This means that our experts saw them as all being important; since infant <6m SAM has been a previously neglected area of research, much is still unknown.

Two questions stood out as having low AEA: “What is the efficacy of providing targeted skilled breastfeeding support to caregivers of stable infants <6m?” and “What are the most feasible tools and techniques for assessing treatment programme coverage for infant <6m SAM?” This implies a lack of consensus about the importance of these questions.

Table 4 shows the same 15 research questions that achieved the highest overall RPS sorted by research category. There was a fairly even spread covering basic epidemiology, health policies, and systems and technical interventions. This illustrated that there are multiple MAMI research priorities across a variety of areas that are not mutually exclusive. It also suggests that the CHNRI method was able to compare and discriminate among research questions from different areas and that there was no systematic bias against questions from any of the three research areas.

**Table 4:** The 15 top research questions ordered according to research instrument (area).

Research instrument	Research question	Q. #	Rank	Answerability	Efficacy	Effective-ness	Deliverability	Sustainability	Disease burden	Equity	RPS	AEA
**Basic epidemiological research**	How should infant <6m SAM be defined?	1	1	94.3	89.5	94.1	98.3	89.7	90.4	91.7	**92.6**	**0.89**
	What are the most feasible tools and techniques for assessing treatment programme coverage for infant <6m SAM?	14	6	89.5	86.1	88.2	100	92.6	84.6	82.0	**89.0**	**0.59**
	How does breastfeeding status and/or change in breastfeeding status impact on infant <6m SAM?	5	13	93.2	90.5	90.9	91.4	82.1	88.5	74.0	**87.2**	**0.79**
	What is the coverage of existing treatment programmes for infant <6m SAM?	13	14	89.3	78.6	84.4	84.5	90.4	81.3	93.8	**86.0**	**0.84**
	How can existing surveys of differing designs and at different levels be adapted to include infant <6m?	7	15	90.0	90.9	81.7	83.9	86.5	73.9	90.5	**85.3**	**0.71**
**Health policy and system research**	What are/is the key opportunities/timing when infant SAM management can be incorporated with other healthcare programmes?	26	2	98.8	90.5	85.9	93.1	90.4	92.0	84.8	**90.8**	**0.79**
	What are the main barriers to existing inpatient interventions for infant <6m SAM and how might they be best addressed?	18	8	93.9	93.1	92.2	87.9	83.9	80.0	89.6	**88.7**	**0.72**
	What is the effectiveness, cost, and safety of an outpatient-focused treatment model for infants with SAM?	21	9	89.2	84.3	93.3	84.5	78.8	93.8	89.6	**87.6**	**0.69**
	Which supervision tools and approaches are most effective towards improving the front-line case management of infant <6m SAM?	27	10	95.0	86.8	89.7	91.4	89.3	84.0	77.1	**87.6**	**0.70**
	How can existing child health and nutrition reporting systems be adapted to capture, monitor and audit data on infant <6m SAM?	28	11	92.7	91.4	80.0	96.6	92.3	83.3	76.1	**87.5**	**0.73**
	What role do CMAM programmes have in delivering outpatient-based treatment for infant <6m SAM?	22	12	90.8	86.4	89.7	78.6	82.7	93.8	89.6	**87.3**	**0.71**
**Technical questions and interventions**	What are the priority components of a package of care for outpatient treatment of infant <6m SAM?	50	3	85.9	92.6	95.0	94.8	89.3	94.0	83.3	**90.7**	**0.68**
	Having detected SAM in the community, what is the efficacy of providing targeted skilled breastfeeding support to caregivers of stable infants?	48	4	86.9	95.6	88.7	87.9	87.5	98.0	88.0	**90.4**	**0.57**
	How can existing tools be adapted and/or linked together to better identify and manage infant <6m SAM?	29	5	91.7	88.9	87.9	91.1	96.2	94.2	81.3	**90.2**	**0.80**
	What is the feasibility, effectiveness, cost-effectiveness and impact of different approaches to promote early initiation and exclusivity of breastfeeding?	58	7	91.3	95.6	86.4	91.4	88.9	86.0	83.3	**89.0**	**0.78**

Table 5 shows the ten research questions that achieved the lowest overall RPS (relative to the other questions). The absolute score for many of these questions was still high. Many of these deal with issues pertaining to a subgroup of malnourished infants <6m, e.g., orphans, infants with disabilities, infants with lactose intolerance and those requiring artificial feeding and/or possible early introduction of complementary feeds. These questions tended to score low on deliverability, effectiveness, and disease burden reduction. Though the bottom questions may not have the greatest overall impact, it does not mean that they are not worth investing in. They are perhaps appropriate for more specialist organisations and funders with more focused interests and priorities.

**Table 5:** The 10 research options that achieved the lowest overall research priority score with average expert agreement.

Research Question	Question number	Rank	Answerability	Efficacy	Effectiveness	Deliverability	Sustainability	Disease burden reduction	Equity	RPS	AEA
What is the feasibility, effectiveness, and cost (effectiveness) of interventions addressing societal and political risk factors of infant <6m SAM?	57	51	61.4	71.0	63.8	62.0	70.8	82.5	90.5	**71.7**	**0.75**
What is the feasibility, validity, and accuracy of using reduced growth velocity to identify, monitor, and discharge infants at high risk of mortality?	30	52	75.7	81.3	78.3	63.0	61.5	70.5	66.7	**71.0**	**0.82**
What is the feasibility, acceptability, and cost (including time) of wet nursing as an option for infants who cannot be breastfed by their mother (e.g., orphans)?	47	53	74.4	77.1	73.1	72.4	65.4	73.1	61.4	**71.0**	**0.78**
What is the efficacy of any supplementary feeds versus exclusive breastfeeding only where this is feasible?	43	54	80.3	75.0	76.6	74.1	68.5	60.0	57.1	**70.2**	**0.74**
What proportion of infant <6m with SAM have underlying disability or other chronic conditions?	4	55	61.4	61.8	57.4	77.8	68.0	64.6	85.7	**68.1**	**0.66**
How can programmes manage artificial feeding so that there is appropriately targeted and adequate support to individual infants without “spilling over” to others?	25	56	69.2	68.2	53.7	73.2	61.1	76.1	69.6	**67.3**	**0.69**
What is the effectiveness and cost (effectiveness) of specialised lactose-free formula during inpatient stabilisation feeds, and how might lactose-intolerant infants be identified?	40	57	78.6	76.7	73.1	64.3	60.5	55.6	57.9	**66.7**	**0.61**
Is there a role for early introduction of complementary feeding before 6 months of age, and in which subgroup of infants would this be indicated?	45	58	67.5	66.7	69.0	63.8	59.3	61.9	69.0	**65.3**	**0.84**
Should there be separate guideline sections such that slightly different interventions may be appropriate for different age groups?	37	59	78.2	76.6	60.7	64.3	58.0	60.4	50.0	**64.0**	**0.73**
What is the feasibility, effectiveness, and cost (effectiveness) of setting up breast milk banking systems in different settings?	19	60	81.9	62.1	51.9	48.1	38.6	52.4	35.7	**53.0**	**0.65**

## Limitations

We acknowledge our study weaknesses. CHNRI attempts to simplify a highly complex process of priority setting, gathering together the opinions of a group of experts to bring coherence and direction to a neglected research area. Some bias is inevitable in the selection of research questions. A longer list of initial questions had to be condensed down to make the survey accessible for as many experts as possible. This was achieved by rephrasing to avoid duplication and repetition of similar concepts; others may have rephrased these questions differently than we did.

Response bias was also likely since those who are interested in infant <6m malnutrition are more likely to respond to a survey on the topic than those who are not. This likely accounts for our relatively low response rate to the questionnaire and is a problem common to all such CHNRI-based assessments. We argue that it is not a serious challenge to the validity of the study since those most interested and familiar with a topic are also in the best position to judge how particular research questions might translate to subsequent practice or policy.

We note that governments and front-line fieldworkers in developing countries were not as well represented (perhaps due to limited internet access to our online survey) as non-governmental organisation (NGO) staff and academics based in industrialised countries. It is possible that these under-represented groups may have answered our questions differently. However, the latter group has particular interest, experience, and expertise in the subject, and so we still believe that their responses are valid.

Unlike some other CHNRI papers, we did not apply weights to our seven judging criteria (according to how the different issues are valued). Our approach is clearer to report, but may not reflect values in practice.

Finally, we focused on management of SAM, an “end point” on a continuum of acute malnutrition. Many of the research questions are applicable to moderate acute malnutrition since the same challenges of how to identify cases and manage them effectively apply. Our focus on SAM in this exercise prioritised those infants <6m with the most risk and also concurs with the target group of the latest WHO guidance.

## Potential Next Steps

Though often expensive, high-quality research is a vital investment towards future health and social services that are effective and cost-effective. Many initiatives aim to improve research design, execution, and reporting so that questions are robustly answered [12,29,30]. More important is to ask the “right” research questions in the first place. We are pleased that our process has highlighted case definitions as the number-one priority for infants <6m. Agreement on this point is vital for the validity and comparability of all other studies. Data already exists, so one ambition is for our CHNRI to catalyse discussions and meetings that will rapidly reach workable consensus on this. This and other prioritisation results are consistent with ten research areas noted by WHO in 2013 (e.g., the need to better define infant <6m SAM; the need to more clearly define therapeutic strategies) [11]. The added value of the CHNRI process and framework is that it gives details and makes clear why certain questions matter and how they might make an impact (e.g., some research questions may be easily answerable but may not make much subsequent impact; others may be challenging (or expensive) to answer but have great potential to improve future intervention effectiveness and sustainability). As well as overall ranking, CHRNI thereby exposes the strengths and weaknesses of numerous potential research options. It is in this that we see other next steps:

- For researchers: our list will support individuals and institutions considering or applying for research funding related to infant <6m SAM. Current focus areas can be compared with those recognised by a wider community. Improving alignment (or justifying differences) between the two will help make a powerful case for investment.
- For funders: our list can be used to help shape grant calls and decide which projects to ultimately fund.

We emphasise that we do not see our list as final or immutable but as a starting point for ongoing dialogue, development, and refinement of research options. As current top questions are answered, others will take their place. Contexts will also change. What will always help, however, is having a framework to guide fair and rational discussions and decisions. Towards this, we believe that the CHNRI approach has a key role to play.

## Conclusions

The management of acute malnutrition in infants <6m (MAMI) is a critical area for child health and nutrition; prioritising research is important for making the biggest advances as quickly as possible. Our results suggest the need for a broad approach spanning basic epidemiology, health policies and systems, and more specialist interventions. Fundamental questions, such as how to define SAM in infants <6m, are most urgent. Other priorities include research on how to integrate MAMI into existing programmes and practices; how to provide appropriate breastfeeding support; and how to assess treatment coverage. The 2013 WHO SAM guidelines create an important stimulus to action. Our CHNRI builds on this by informing global efforts and providing technical direction on an agreed-on and transparent operational research agenda: maximising opportunities, avoiding duplication of effort and waste of resources, and designing research that will most effectively inform policy change and front-line practice. All ultimately lead to MAMI treatments with the biggest possible impact on infant <6m mortality, morbidity, and nutrition.

## Supporting Information

S1 Table Full results table. (XLSX)

S1 Text Details of the CHNRI methodology. (DOCX)

## Abbreviations

AEA: average expert agreement
CHNRI: Child Health and Nutrition Research Initiative
CMAM: Community-Based Management of Acute Malnutrition
DALY: disability-adjusted life year
MAM: Moderate Acute Malnutrition
MAMI: Management of Acute Malnutrition in Infants aged less than six months
MDG: Millennium Development Goal
MUAC: mid-upper arm circumference
NGO: non-governmental organisation
NUGAG: Nutrition and Growth Advisory Group (WHO)
RPS: research priority score
SAM: Severe Acute Malnutrition
SUN: Scaling Up Nutrition
UN: United Nations
WHO: World Health Organization
