# Supplementary material for: Research Priorities to Improve the Management of Acute Malnutrition in Infants Aged Less Than Six Months (MAMI)
Source: PLoS Med. 2015 Apr 21;12(4):e1001812. doi: 10.1371/journal.pmed.1001812 (PMC4405387; doi:10.1371/journal.pmed.1001812)
Supplement: S1 Text — (DOCX) [file pmed.1001812.s002.docx]

### **SUPPORTING TEXT**

The below sections are based directly on other CHNRI reports, <http://www.chnri.org/publications.php> . Sections a and b have been adapted for this particular exercise on infant <6m malnutrition. Sections c and d, which are standard for every CHNRI are however copied direct from other publications since we repeated the same standard method.

### **Defining the context and criteria**

### Specifying the context is the important first step of the CHNRI process. The context for this exercise was defined as follows:

- **Burden of disease of interest**: Infant Severe Acute Malnutrition (SAM)
- **Population of interest:** Infant <6month in low or middle income countries
- **Existing policy/target:**
  - **Global context**: reduction of infant (under one) mortality rate and under-five mortality rate by two thirds by 2015 (MDG 4). Halve the prevalence of underweight children under five years of age (MDG 1)
  - **Specific context:** Following an expert consultation (NUGAG meeting, Nutrition and Growth advisory group, WHO, Feb 2012), WHO guidelines of the management of severe acute malnutrition will for the first time include infants aged <6months. The meeting recognised paucity of evidence related to this group. This has created a need for research, and in particularly for prioritised research.
- **Level of urgency**:
  - **Global context:** high, because the MDG goals are not being achieved
  - **Specific context:** high, because forthcoming WHO guidelines are currently based on low quality evidence as that is all that is available at present
- **Time Frame**: to achieve detectable improvements in management of infant <6month SAM by 2015/2020/beyond

1. **Choice of technical experts and systematic listing and scoring of research options**

A group of experts who had contributed to the original MAMI (Management of Acute Malnutrition in Infants) project or related work were asked to lead the CHNRI process – the MAMI CHNRI “Core Group” of authors. Both during the original project and in subsequent meetings and discussions, a large number of research options had emerged, many of which were described in the MAMI project report. Other key meetings where concepts for research options arose:

- WHO NUGAG meeting in Geneva, February 2012
- Staff consultation facilitated by Action Contre La Faim (ACF) in Paris, February 2012
- The Infant Feeding in Emergencies (IFE) Core Group meeting in Oxford, March 2012
- An Infant and Young Child Feeding (IYCF) meeting facilitated by UNICEF/The Global Nutrition Cluster/Save the Children UK in London, June 2012.

From a long initial list of research options, we refined and narrowed down to what was felt to be a manageable number for the scorers. This was achieved by careful phrasing and consolidation of questions to limit the overlap between proposed ideas. The final number of research questions was 60, divided into three categories: (1) Basic epidemiological research; (2) Health policy and systems research and (3) Technical questions and interventions. Based on CHNRI’s conceptual framework, we also agreed on seven judging criteria: answerability; efficacy; effectiveness; deliverability; sustainability; disease burden reduction; equity. Please see table 1 in the main paper for details of how we asked applied each of these criteria to each of the 60 research options.

The 60 research options, together with the 7 questions for each option were formatted into an online survey, using ‘Survey Monkey’ ([www.surveymonkey.com](http://www.surveymonkey.com)). The survey was ordered so that scorers judged all questions by each judging criterion in turn. The survey was divided into two parts.

- Part 1 included the following criteria: answerability, efficacy and effectiveness;
- Part 2 included: deliverability, sustainability, disease burden reduction and equity.

The reason for this split was to maximise response rate: shortening the survey for any one individual and hence making it more ‘doable’ and more likely to be filled in rather than abandoned part way through.

A wider group of experts was invited to score the research questions. These were drawn from existing databases of those who had contributed to the original MAMI project and/or attended related meetings (the Emergency Nutrition Network is a “hub organization in the field of nutrition and infant/young child feeding). Individuals from these lists represented a breadth and depth of expertise in research, policy making and/or programme management. Every effort was made to invite a mix of individuals from different professional backgrounds (epidemiologists, clinicians, public health professionals and programme managers) and from different regions, to encourage a diversity of viewpoints. 150 experts were invited to participate in this way and were asked to complete either part 1 or part 2 of the survey at random. An open invitation was also posted on several relevant discussion forums online: CMAM forum (<http://www.cmamforum.org/>); Emergency Nutrition Network, ENN (<http://fex.ennonline.net/43/mami>). Here, participants were asked to flip a coin to decide whether to participate in part 1 or part 2. Once participants completed their randomly allocated part 1 or part 2 they were also invited to complete the other part if time allowed. All participants were automatically entered into a prize draw for TALC (Teaching Aids at Low Cost) vouchers to provide an added incentive to participate. The entire process was conducted and completed within six weeks between September and December 2012.

64 individuals participated in the survey. These individuals comprise the ‘MAMI Research Group’, named at the end of the paper. Seven others also took part anonymously. 34 completed part 1 only, 20 completed part 2 only and ten completed both parts.

1. **Computation of "Research Priority Scores" (RPS)**

All the experts answered the questions listed in Table 1a by ‘Yes’ (1 point), ‘No’ (0 points), ‘Undecided’ (0.5 points) or ‘Insufficiently informed’ (missing input).

Each research option was scored for each of the seven criteria excluding the missing input, i.e. as the proportion of maximum possible points scored when an answer was given. Each of the 60 research options received seven scores, one for each judging criteria, each ranging between 0% (lowest possible priority score) to 100% (maximum possible priority score). The overall research priority score (RPS) was calculated as the mean of all seven priority scores. The final list of priorities with intermediate and final priority scores for all 60 research options is presented in Table 2**.** The list of technical experts who participated is presented in the “Group Author list”.

1. **Assessment of agreement between scorers (AEA)**

As well as the overall priority given to various research options, CHNRI methodology also assesses the level of agreement between scorers in order to expose issues of greatest agreement and controversy. This is calculated for each scored research option as:

$$AEA=\frac{1}{7} ₓ\sum_{q=1}^{7} \left( . \frac{N \left( of scorers who provided the most frequent response \right)}{N of all scorers who provided a response} \right)$$

where q is a question that experts are being asked to evaluate each competing research investment option again, ranging from 1 to 7.

An AEA score of 0 would mean no agreement at all; 1 would mean perfect agreement between all respondents.
